# Effects of an Intermittent Grape-Seed Proanthocyanidin (GSPE) Treatment on a Cafeteria Diet Obesogenic Challenge in Rats

**DOI:** 10.3390/nu10030315

**Published:** 2018-03-07

**Authors:** Iris Ginés, Katherine Gil-Cardoso, Joan Serrano, Àngela Casanova-Martí, MTeresa Blay, Montserrat Pinent, Anna Ardévol, Ximena Terra

**Affiliations:** MoBioFood Research Group, Departament de Bioquímica i Biotecnologia, Universitat Rovira i Virgili, 43007 Tarragona, Spain; iris.gines@urv.cat (I.G.); katherine.gil@urv.cat (K.G.-C.); joan.serrano@urv.cat (J.S.); angela.casanova@urv.cat (À.C.-M.); mteresa.blay@urv.cat (M.B.); montserrat.pinent@urv.cat (M.P.); ximena.terra@urv.cat (X.T.)

**Keywords:** proanthocyanidins, dose, cafeteria-diet, rat, metabolic syndrome

## Abstract

Obesity is highly associated with the pathologies included in the concept of the Metabolic Syndrome. Grape-seed proanthocyanins (GSPE) have showed very positive effects against all these metabolic disruptions; however, there is, as yet, no consensus about their effectiveness against an obesogenic challenge, such as a cafeteria diet. We determined the effectiveness of a dose of 500 mg GSPE/kg b.w. (body weight) against the obesogenic effects of a 17-week cafeteria diet, administered as a sub-chronic treatment, 10–15 days before, intermittently and at the end of the diet, in Wistar rats. Body weight, adiposity, indirect calorimetry and plasma parameters were analyzed. GSPE pre-treatment showed a long-lasting effect on body weight and adiposity that was maintained for seven weeks after the last dose. A corrective treatment was administered for the last two weeks of the cafeteria diet intervention; however, it did not effectively correct any of the parameters assessed. The most effective treatment was an intermittent GSPE dosage, administered every second week during the cafeteria diet. This limited body weight gain, adiposity and most lipotoxic effects. Our results support the administration of this GSPE dose, keeping an intermittent interval between dosages longer than every second week, to improve obesogenic disruptions produced by a cafeteria diet.

## 1. Introduction

Obesity is a health epidemic affecting more than 20% of the Western population, and its incidence is steadily increasing [1]. Excessive body weight significantly increases the risk and prognosis of metabolic syndrome (diabetes mellitus type 2, cardiovascular disease, hyperlipidemia, nonalcoholic fatty liver disease) and several types of cancer [2].

Obesity and being overweight are multifactorial problems that require more knowledge to identify their origin and determine the best solutions to reduce their incidence. However, lifestyle interventions, specifically, intensive counseling programs on diet and physical activity which aim to reduce energy intake and increase energy expenditure, are the main strategies currently recommended for preventing obesity [2]. There are some drugs that act on these targets but the most recent advances in food and nutrition sciences highlight the concept of modulating food intake and/or energy expenditure through food design [3]. Therefore, complementary dietary strategies, such as bioactive compounds with anti-obesity effects, could be an adjunctive support to current therapies and reinforce obesity treatments. 

Proanthocyanidins (PACs), which have been shown to have many healthy properties, are interesting candidates as components of functional food ingredients [4]. PACs are a group of polyphenols widely distributed in nature, and in fruits and vegetables and their beverage products, such as red wine and tea. In general, the action mechanisms regarding the anti-obesity effects of PACs appear to be associated with increasing energy expenditure [4]. PACs cause the upregulation of energy expenditure-related genes in skeletal muscle and the liver whilst decreasing fatty acid synthesis and fat uptake in the liver [5]. At the gastro-intestinal level, some authors have found that they play a role as inhibitors of some digestive enzymes, such as lipase and amylase, repressing fat and glucose absorption from the gut [6] and modulating enteroendocrine secretions [7,8] that could affect satiety [9]. PACs could also play a preventive role in obesity due to their effects on inflammatory mediators and cellular events that modulate inflammation and adipose tissue dysfunction, and/or their effect on microbiota [10]. The effects of PACs on body weight have been reviewed [4], and some discrepancies in the literature have been found. PACs decreased body weight gain in several experiments; however, in other experiments, no effects were observed. This dichotomy emphasizes the fact that the dosing strategies and physiological conditions need to be optimized before an anti-obesity effect can be determined. From a functional food perspective, the most appropriate way for a compound to be effective as an anti-obesity agent is to prevent and/or treat the problem at the initial stages. In this sense, we have previously reported that a sub-chronic treatment with a grape-seed extract rich in proanthocyandins (GSPE) at a dose of 500 mg/kg of body weight was effective for reducing body weight by limiting food intake and activating energy expenditure in rats on a standard chow diet [9]. To prove their effectiveness under an obesogenic situation, in the present study, we evaluated the effects of this dose of GSPE (500 mg/kg), administered at different time points with respect to the start of the obesogenic challenge. With this experimental design, we aimed to compare the effectiveness of different dosing strategies against excessive body weight gain and homeostatic disarrangements associated with the metabolic syndrome.

## 2. Materials and Methods

### 2.1. Proanthocyanidin Extract

The grape seed extract enriched in proanthocyanidins (GSPE) was kindly provided by *Les Dérivés Résiniques et Terpéniques* (Dax, France). According to the manufacturer, the GSPE composition used in this study (batch number: 124029) contains monomers of flavan-3-ols (21.3%), dimers (17.4%), trimers (16.3%), tetramers (13.3%) and oligomers (5–13 units; 31.7%) of proanthocyanidins. A detailed analysis of the monomeric to trimeric structures can be found in the work of Margalef and colleagues [11].

### 2.2. Animal Models

**Figure 1:**
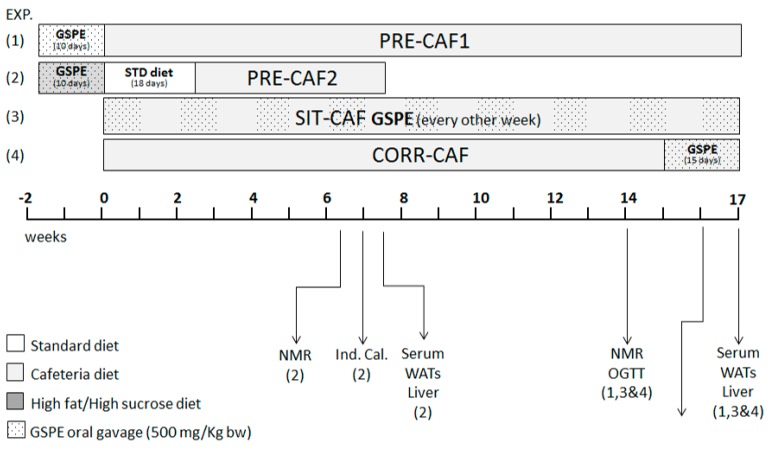
Schematic diagram of the experimental design. CAF: cafeteria diet; (1) PRE-CAF1: rats receiving GSPE (Grape-seed proanthocyanidins) preventive treatment 10 days before the cafeteria intervention started; (2) PRE-CAF2: rats receiving GSPE preventive treatment for 10 days together with a high fat/high sucrose diet followed by the cafeteria diet intervention; (3) SIT-CAF: rats receiving GSPE treatment simultaneously and intermittently with the cafeteria diet every other week; (4) CORR-CAF: rats receiving GSPE corrective treatment during the last 15 days of the cafeteria intervention. GSPE: grape seed proanthocyanidin extract; NMR: nuclear magnetic resonance; OGTT: oral glucose tolerance test; Ind. Cal.: Indirect calorimetry; WATs: white adipose tissues.

Two parallel studies were performed for this experiment: a long-term challenge study with fifty Wistar rats and a short-term challenge study with fourteen Harlan Rcc: Han rats, detailed in Figure 1. 

All animals were female rats, each weighing 240–270 g; they were purchased from Charles River Laboratories (Barcelona, Spain). After one week of adaptation, the rats were individually caged in the animal quarters at 22 °C with a 12-h light/12-h dark cycle and were fed *ad libitum* with a standard chow diet (Panlab 04, Barcelona, Spain) and tap water. After a period of acclimation, the animals were randomly distributed into the experimental groups (*n* = 7–10) and were fed a standard chow diet *ad libitum* for the whole duration of the experiment. The control group (STD) only received the standard chow diet. The rest of the groups, in addition to the standard chow, received a cafeteria diet, as a model of a high fat/high sucrose diet and/or a GSPE supplement for different periods. The cafeteria diet consisted of bacon, sausages, biscuits with paté, carrots, muffins, and sugared milk, which induces voluntary hyperphagia [12]. Table 1 summarises energy contents of meals offered each group. This diet was offered freshly *ad libitum* every day to the animals, with enough food for either 5 weeks (short-term challenge) or 17 weeks (long-term challenge).

In the long challenge, apart from the standard (STD) and the cafeteria (CAF) group, there were three more groups that received the cafeteria diet plus an oral GSPE supplementation at a dose of 500 mg GSPE/kg b.w.( Body Weight): (1) 10 days before the long-term cafeteria intervention started as a preventive treatment (PRE-CAF1); (2) simultaneously with the long-term cafeteria diet every other week (Simultaneous-Intermittent-Treatment-CAF; SIT-CAF); and (3) during the last 15 days of the long-term cafeteria intervention as a corrective treatment (CORR-CAF). GSPE was dissolved in water and was orally gavaged to the animals at 18:00 h for each treatment in a volume of 500 µL, one hour after removing all available food. The animals that were not supplemented with GSPE received water as a vehicle.

There were two groups of animals in the short-term challenge: CAF and PRE-CAF2. Both groups received a simplified high-fat-high-sucrose diet for the first 10 days of the assay, consisting of a palatable hypercaloric emulsion presented in an independent bottle, containing (by weight) 10% powdered skimmed milk, 40% sucrose, 4% lard and 0.35% xanthan gum as a stabilizer [13]. The PRE-CAF2 group received the GSPE dose during these ten days as defined previously. Afterwards, all the animals were kept for 18 days (≈3 weeks) on a standard chow diet. At this point in time, all these rats started with the short-term CAF diet challenge.

### 2.3. Blood and Tissue Collection

At the end of the study, animals were fasted for 1–4 h, anesthetized with sodic pentobarbital (70 mg/kg body weight; Fagron Iberica, Barcelona, Spain) and exsanguinated from the abdominal aorta. The blood was collected using heparin (Deltalab, Barcelona, Spain) as an anticoagulant. Plasma was obtained by centrifugation (1500× *g*, 15 min, 4 °C) and stored at −80 °C until analysis. The different white adipose tissue depots (retroperitoneal (rWAT), mesenteric (mWAT) and periovaric (oWAT)), brown adipose tissue (BAT), liver, kidneys, spleen and thymus were rapidly removed and weighed.

In the long-term study, blood samples were obtained from overnight fasted animals at week 14 to check their insulin-resistance by the Homeostatic Model Assessment for Insulin Resistance (HOMA-IR) [14].

All the procedures were approved by the Experimental Animal Ethics Committee of the Universitat Rovira I Virgili (code: 0152S/4655/2015).

### 2.4. Morphometric Variables

Body weight was monitored weekly and body composition was measured in conscious rats at indicated times by nuclear magnetic resonance (NMR) imaging (EchoMRI 2004, Echo Medical Systems, Houston, TX, USA), which provided total body fat and lean mass data.

Adiposity was expressed through two adiposity indexes, which were based on the total fat pads obtained [15] or the NMR measurements.

### 2.5. Biochemical Variables

Colorimetric enzyme commercial kits were used to measure plasma glucose (QCA, Amposta, Spain), non-esterified fatty acids (NEFAs) (Wako, Neuss, Germany), and triacylglycerol (TAG) (QCA, Amposta, Spain) levels. Insulin levels were analyzed with a rat insulin ELISA kit (Mercodia, Uppsala, Sweeden). Plasma Tumor Necrosis Factor-α (TNF-α) levels were measured with rat ELISA kits (Millipore, Darmstadt, Spain, and Abcam, Cambridge, UK, respectively). Hepatic and plasma triglycerides were assayed according to Quesada and col [16], and plasma urea was analyzed with a colorimetric kit (QCA, Amposta, Spain).

### 2.6. Indirect Calorimetry

The respiratory metabolism was measured in the animals in the last week of each study using a ventilated hood system (Panlab Harvard Apparatus, Barcelona, Spain). For this purpose, animals were transferred from their cages to an acrylic box from 08:00 a.m. to 02:00 p.m. After an initial acclimatization period of 1 h, oxygen consumption (VO_2_) and carbon dioxide production (VCO_2_) were measured every 9 min over a period of 5 h by an O_2_ and CO_2_ analyzer at a controlled flowrate of 600 mL/min. At each point of the analysis, the software program Metabolism 2.1.02 (Panlab Harvard Apparatus, Barcelona, Spain) automatically calculated the respiratory quotient (RQ) as the VCO_2_/VO_2_ ratio and the Energy Expenditure (EE) in kcal/day/kg^0.75^ as VO_2_ × 1.44 × [3.815 + (1.232 × RQ)], according to the Weir formula [17].

### 2.7. Statistical Analysis

The data are represented as the mean ± standard error of the mean (SEM). Statistical comparisons between groups were assessed by ANOVA, followed by Tukey post-hoc tests. Analyses were performed with XLStat 2017.01 (Addinsoft, Barcelona, Spain). *p*-Values < 0.05 were considered statistically significant.

## 3. Results

### 3.1. GSPE Limits Body Weight Gain under Obesogenic Diets When Administered as a Preventive Agent

To identify the best time-point for administering the GSPE dose assayed (500 mg/kg b.w.) against the unhealthy effects of an obesogenic diet, we first tested its effectiveness on body weight gain and adiposity after different administration periods.

**Figure 2:**
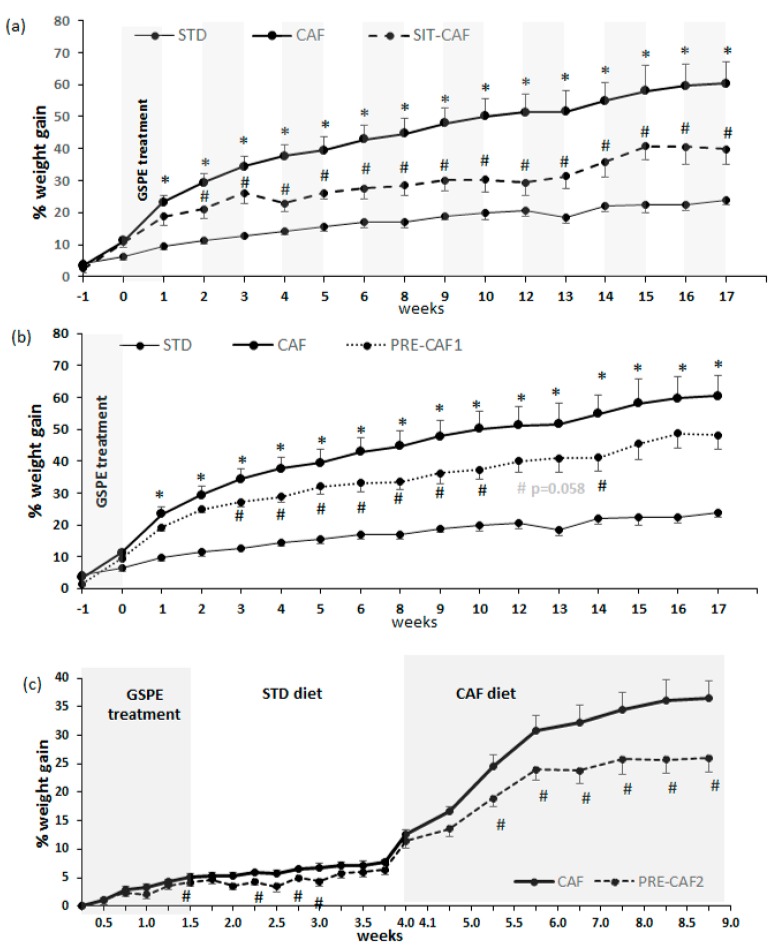
Body weight gain during the experiment. Body weight was measured weekly throughout the whole experiment. STD: lean rats fed a standard chow diet; CAF: rats fed a cafeteria diet; PRE-CAF1: rats receiving GSPE preventive treatment 10 days before the cafeteria intervention started; PRE-CAF2: rats receiving GSPE preventive treatment for 10 days together with a high fat/high sucrose diet followed by the cafeteria diet intervention; SIT-CAF: rats receiving GSPE treatment simultaneously and intermittently with the cafeteria diet every other week. Values are means ± SEM. * *p* < 0.05 compared to STD rats. ^#^ *p* < 0.05 compared to the CAF group.

Figure 2a shows that the ingestion of a cafeteria diet led to a significant increase in body weight gain from the 1st week of treatment until the end (17 weeks). The final body weight in the cafeteria-fed animals was markedly greater compared to standard chow-fed animals (Table 2). The SIT-CAF treatment, with GSPE administered every other week, led to a significant reduction in body weight gain from the second week until the end of the experiment, when it reached a 14% reduction with respect to the CAF group (Figure 2a). Table 2 shows that final body weight in this GSPE treated group was significantly lower than in cafeteria-fed rats. Figure 2b shows that a GSPE pretreatment for only 10 days before the beginning of the cafeteria-diet intervention (PRE-CAF1) led to a lower increase in body weight, which was statistically significant from the 3rd to the 14th week. This treatment limited body weight gain by 9%, although at the end of the experiment this parameter was not statistically different from cafeteria-fed animals. As this long-lasting effect has not been previously defined, we analyzed it further under other dietary conditions (short-term study, PRE-CAF2). The administration of GSPE together with a simplified high fat/high sucrose diet for 10 days (PRE-CAF2) limited the final body weight (g: 231 ± 0.97 vs. 237 ± 1.65; *p* = 0.011). Interestingly, when PRE-CAF2 animals were returned to a standard diet, the effect of the GSPE pretreatment on body weight gain was maintained for more than one week after the GSPE treatment finished and was evident between days 14 and 22 (Figure 2c). Moreover, when the rats were later challenged with a cafeteria diet for 5 weeks, the GSPE was still able to markedly and consistently limit body weight gain until the end of the experiment (Figure 2c). The body weight gain at the end of this PRE-CAF2 treatment was 26% vs. 37% in the cafeteria group (Figure 2c), which is similar to the reduced body weight gain obtained at the end of the long-term preventive approach (32% PRE-CAF1 vs. 40% CAF group; Figure 2b).

Finally, we also tested GSPE supplementation as a corrective treatment. Animals received the GSPE dose during the last two weeks of the cafeteria diet (16th and 17th weeks). In this case, GSPE treatment led to a slight (4% with respect to the CAF group), but non-significant, reduction in final body weight (Table 1).

### 3.2. Adipose Tissue is an Important Target of GSPE Preventive Effects against Obesogenic Diets

Once it had been demonstrated that some GSPE interventions were able to modulate body weight gain, we analyzed the body composition of the animals. We used NMR to measure the total fat mass of each animal at two time points: week 8 for the short-term intervention experiment (Table 3) and week 14 for the long-term intervention (Table 2). The results show the same profile found previously for body weight. The SIT-CAF group showed significantly reduced total fat masses, and PRE-CAF2, 7 weeks after the last GSPE dose, also showed a clear statistically-significant reduction in total adiposity. Fourteen weeks after the GSPE treatment, there was a tendency to maintain reduced fat levels compared to the CAF group in the PRE-CAF1 group, but it was not statistically different.

To analyze whether the GSPE effect is specific for some adipose tissues, we weighed the different adipose depots. As expected, visceral adiposity increased due to the cafeteria diet compared to STD rats (Table 2). The SIT-CAF GSPE supplementation significantly reduced the weight of all the visceral white adipose tissue depots weighted (periovaric, mesenteric and retroperitoneal). The animals pre-treated with GSPE for 10 days showed a significant reduction in adipose tissue depots after 5 weeks of a cafeteria diet (PRE-CAF2, Table 3). However, after 17 weeks of cafeteria-diet feeding (PRE-CAF1), the reduction in adipose tissue was not statistically significant (Table 2). In PRE-CAF1, only the mesenteric and retroperitoneal WAT weights were lower than the CAF group. Pre-treatment with GSPE showed similar BAT weight lowering effects after 5 weeks of cafeteria diet treatment (PRE-CAF2, Table 3), although there was no difference after 17 weeks of the cafeteria diet (PRE-CAF1, Table 2).

Table 2 shows the ineffectiveness of the corrective treatment (CORR-CAF) against adiposity indexes at the end of the treatment.

Finally, the weights of the main metabolically active tissues were also measured in the animals after the long-term treatments (Table 2). The liver, thymus and kidney weights were increased by the CAF diet, and no GSPE treatment affected the weights of these organs. Spleen and pancreas weights were unchanged by either the cafeteria diet or the GSPE treatments.

### 3.3. All GSPE Treatments Reduced RQ but Did Not Modify Oxygen Expenditure

To further analyze differences between treatments on the whole organism energetics, we conducted indirect calorimetry measurements 1 week before sacrifice. To discard any effects on protein metabolism, we measured the amount of urea in the plasma (mM; STD: 4.9 ± 0.11; CAF: 4.7 ± 0.3; PRE-CAF1: 4.5 ± 0.14; SIT-CAF: 4.2 ± 0.37; CORR-CAF: 4.7 ± 0.15) and observed no significant differences between groups.

**Figure 3:**
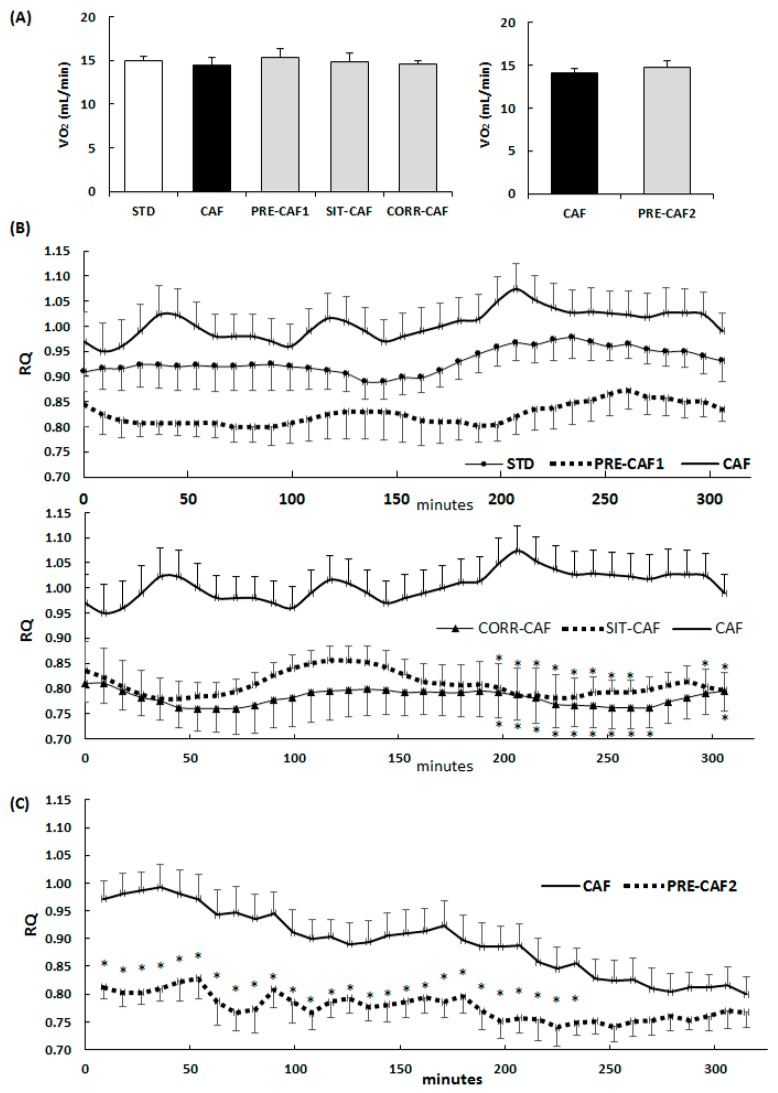
Patterns of energy expenditure and Respiratory Quotient (RQ) due to GSPE-treatments. Rats entered an indirect calorimeter for respiratory measurements at 08:00 am and remained until 02:00 p.m. Short-term challenge measurements were taken during the 7th week of the post-GSPE treatment (*n* = 7 animals/ group). Long-term challenge measurements were taken on the first day of the 17th week of the cafeteria diet (*n* = 6 animals/ group). (**A**) Mean oxygen consumption measured (long-term challenge in the left panel and short-term challenge in the right panel); (**B**) Mean RQ measured in the long-term challenge (upper panel, effects due to PRE-CAF1 treatments; lower panel, effects on animals in SIT-CAF treatment); (**C**) Mean RQ measured in the short-term challenge for PRE-CAF2 treatment vs. CAF. STD: standard chow diet; CAF: cafeteria diet; PRE-CAF1: rats receiving GSPE preventive treatment 10 days before the cafeteria intervention started; PRE-CAF2: rats receiving GSPE preventive treatment for 10 days together with a high fat/high sucrose diet followed by a cafeteria diet intervention; SIT-CAF: rats receiving GSPE treatment simultaneously and intermittently with the cafeteria diet every other week; RQ: respiratory quotient. Values are means ± SEM. * *p* < 0.05 compared with CAF group.

Figure 3A shows the mean oxygen consumption in the five hours after the light-period started, which was similar in all the groups and treatments. Figure 3B shows that the RQ values of the chow diet group and the cafeteria animals were not different, which could be expected based on the RQ estimated from the macronutrient composition of the diets (0.96 for the cafeteria diet and 0.98 for the standard chow). Interestingly, the incorporation of GSPE at any time point of the study decreased the RQ towards a higher oxidation of lipidic substrates, as can be seen in Figure 3 (PRE-CAF1, SIT-CAF and CORR, Figure 3B and PRE-CAF2, Figure 3C).

### 3.4. GSPE Pretreatment Prevents the Development of Metabolic Syndrome in Rats Fed a Cafeteria Diet

Because obesity is associated with several metabolic disturbances, such as dyslipidaemia, inflammation and insulin resistance, in addition to morphometric variables, we also examined the metabolic state of the animals after the dietary challenge.

Table 2 shows the effectiveness of the SIT-CAF treatment in reducing plasma TAG and cholesterol at the end of the experiments. None of the other treatments produced statistically significant effects on the plasma lipid parameters (Table 2 and Table 3).

**Figure 4:**
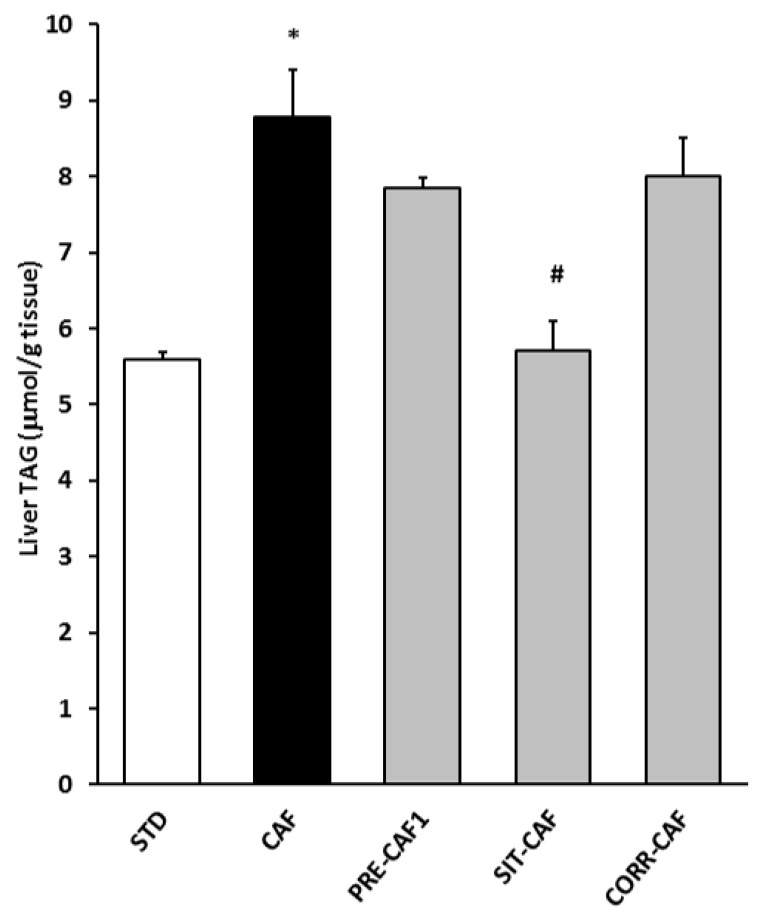
Liver triglyceride reduction after simultaneous and intermittent (SIT-CAF) treatment with GSPE. STD: lean rats fed a standard chow diet; CAF: rats fed a cafeteria diet; PRE-CAF1: rats receiving GSPE preventive treatment 10 days before the cafeteria intervention started; SIT-CAF: rats receiving GSPE treatment simultaneously and intermittently with the cafeteria diet every other week; CORR-CAF: rats receiving GSPE corrective treatment during the last 15 days of the cafeteria intervention; TAGs: triglycerides. Values are means ± SEM. * *p* < 0.05 compared to STD rats. # *p* < 0.05 compared to the CAF group.

In addition, an important feature of the metabolic syndrome associated with obesity is the impaired lipid storage capacity of white adipose tissues, which is related to ectopic fat accumulation in the liver, triggering hepatic steatosis. When we evaluated hepatic lipid accumulation, we found that, as expected, the CAF diet induced a marked increase in TAG accumulation in the liver and that the GSPE SIT-CAF treatment completely prevented this (Figure 4). None of the other treatments produced statistically significant effects. However, the PRE-CAF2 treatment showed an increased liver TAG content compared to the control treatment (CAF = 8.8 ± 0.35; PRE-CAF2 = 9.8 ± 0.21 mmol/g of liver; *p* = 0.05).

GSPE treatment did not result in any significant differences in glucose or insulin levels either after 17 weeks of treatment (Table 2) or in the short intervention study (PRE-CAF2). Since the animals were sacrificed in a non-fasted state, we evaluated insulin resistance at week 14, after an overnight fast period. The results obtained showed no statistically significant differences between the groups analyzed, although HOMA-IR tended to be higher in CAF (2.5 ± 0.5) compared to STD (1.3 ± 0.1; *p* = 0.075), and it tended to be lower in the SIT-CAF treated rats (SIT-CAF: 1.2 ± 0.1) compared to the CAF group (*p* = 0.079).

**Figure 5:**
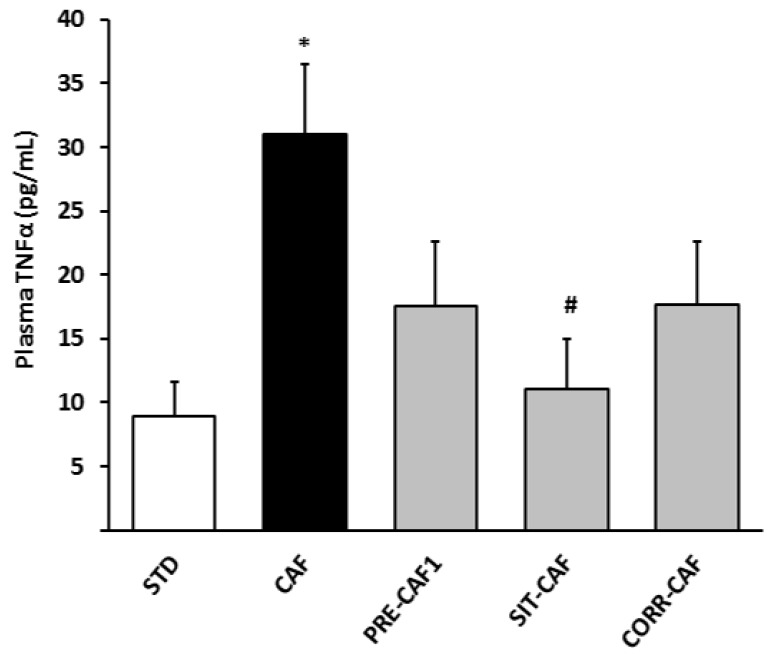
Amelioration of TNF-α plasma levels in GSPE treated groups. STD: lean rats fed a standard chow diet; CAF: cafeteria diet; PRE-CAF1: rats receiving GSPE preventive treatment 10 days before the cafeteria intervention started; SIT-CAF: rats receiving GSPE treatment simultaneously and intermittently with the cafeteria diet every other week; CORR-CAF: rats receiving GSPE corrective treatment during the last 15 days of the cafeteria intervention; TNFα: tumor necrosis factor-alpha. Values are means ± SEM. * *p* < 0.05 compared to STD rats. # *p* < 0.05 compared to the CAF group.

Finally, we evaluated TNF-α levels as an indicator of systemic inflammation. Figure 5 shows that 17 weeks of a CAF diet induced a three-fold increase in TNF-α levels compared to STD rats, and that the SIT-CAF treatment significantly reduced TNF-α levels. PRE-CAF1 rats did not show a protective effect at 17 weeks. After 5 weeks of a cafeteria diet, there was no clear increase in TNF-α levels, and it was not possible to evaluate the effects of GSPE on it.

## 4. Discussion

Grape-seed derived proanthocyanidins have been proven to have lipolytic properties [4]. There is also a high consensus regarding their abilities to improve cholesterol metabolism [5] and work as anti-inflammatory [18] and anti-hyperglicemic [19] agents. Their properties against hypertension have also been described [20,21]. In summary, these properties make them good candidates for being agents against metabolic syndrome. However, not all diet formulations, animals and times of administration show the same effects. Here we have assessed their effects on most of these metabolic risk factors with a GSPE dose that has been previously proven to inhibit food intake in chow-fed animals [9]. Our results support the proposal that grape-seed derived proanthocyandins play an effective role as preventive agents against obesogenic-induced damage that is highly related to their lipolytic properties.

CAF-diet fed rats exhibited voluntary hyperphagia that resulted in dramatic and rapid body weight gain, and thus they developed a representative model of the human metabolic syndrome [12]. The daily dose of 500 mg GSPE/kg of b.w. over 10 days was proven to effectively limit body weight gain under a chow diet in male rats [9] and female rats [22]. At the end of the treatment, this effect was reproduced in the present study in the PRE-CAF1 group. This study shows the effectiveness of GSPE in impeding body weight gain 10 days after its administration, together with a moderately palatable diet, as shown by the PRE-CAF2 group. In contrast, the same treatment applied to already obese animals (after 15 weeks of cafeteria diet) did not show clear effects on body weight, as shown by the CORR-CAF treatment. The effects of GSPE on adipose depots paralleled the results on body weight. As already stated, GSPE has been demonstrated to be a lipolytic agent [4,23,24], and all the assessed GSPE treatments were associated with reduced RQs during the light period measured, under the same cafeteria diet. This higher lipolysis should limit adipose accrual, as the preventive effect clearly showed during the first weeks after treatment in the two PRE-CAF approaches. However, this lipolytic effect was not enough to result in statistically significant differences in adipose accumulation when the adipose accrual was already too high, which was the case for the CORR-CAF treatment. Our results suggest that GSPE is effective on adipose accrual depending on the amount of adipose depot there is at the start of the treatment. Other authors have analyzed similar short-term corrective treatments with GSPE in dietary doses (25–50 mg GSPE/kg b.w./day). After 2 weeks of a cafeteria diet in hamsters, GSPE treatment for 15 days was proven to effectively correct body weight gain [25]. However, GSPE treatment was not effective after 8 weeks of a cafeteria diet and a posterior corrective treatment of 3 weeks with 25 mg GSPE/b.w., nor after 13 weeks of a cafeteria diet in rats (10 or 20 days with doses of 25 or 50 mg GSPE/kg b.w.) [26]. All of these studies show that GSPE has a corrective effect on body weight adiposity when the adipose accrual is moderate. There is more consensus in studies involving the effectiveness of dietary doses on limiting body weight and adiposity when proanthocyanidins have been administered in a preventive manner from the beginning of the obesogenic diet [27,28]. Working with doses around 500 mg GSPE/kg b.w., a non-dietary dose, we showed that a dose of least 350 mg GSPE/kg b.w. is necessary to obtain satiating effects simultaneously to lipolytic action after 8–10 days of subchronic treatment [29]. Bao and colleagues proposed a minimum dose of 250 mg/kg, administered for 16 weeks, to obtain effects on body weight and also satiety, in a Diabetes Mellitus 1 model [30]. Recently, the antiadipogenic effect of a 300 mg/kg b.w. dose in conjunction with a high fat diet for 7 weeks has also been proven [31]. Thus, from the analysis of all these effects, it could be said that GSPE is effective under moderate obesogenic conditions, and it has a greater effect as a preventive agent when it is administered from the beginning of the obesogenic diet.

Our study describes, for the first time, a long-lasting effect on body weight gain that has not been previously shown for proanthocyanidins. We showed that the effects of GSPE on body weight and adiposity were maintained for several weeks after GSPE administration had finished, even with a very obesogenic diet like the cafeteria diet. The two pre-treatment studies both showed, in the seven weeks after a 10-day treatment with 500 mg GSPE/kg b.w., that there was a lasting effect on body weight that remained until the 14th week. This is a very novel effect that has not been previously shown for this parameter. It opens the way for new administration formulas distributed in time, such as, for example, the SIT-CAF approach tested in this work. The administration of this dose every other week resulted in the most effective treatment of those assessed: it limited body weight gain induced by the cafeteria diet by 50% and adipose accumulation by 60%. This intermittent treatment resembles improvements found in laboratory rats and mice kept on intermittent fasting (IF) diets, which includes eating patterns in which individuals go for extended periods of time (e.g., 16–48 h) with little or no energy intake, with intervening periods of normal food intake on a recurring basis, revised by Mattson and colleagues [32]. The responses to IF include reduced body fat, reduced levels of insulin and leptin that parallel increases in insulin and leptin sensitivity and reduced inflammation. Another similar effect between the IF diet [33] and SIT-CAF GSPE is that they do not affect lean body mass (results not shown). In fact, none of the GSPE treatments assessed changed this.

There are two aspects to consider when analyzing the effects of the different GSPE treatments on the metabolic disruptions produced by the cafeteria diet. The higher tendency for increased lipid oxidation following GSPE suggests a trend towards limiting the lipotoxicity origins of metabolic syndrome-related pathologies [34]. However, it can be clearly seen that the time when it is administered in relation to the time-course of a cafeteria diet is also very important [35]. Body weight gain and adiposity are clearly found after 5 or 8 weeks of a cafeteria diet [36]. Hypertriglyceridemia has been observed from the 4th week of a cafeteria diet in hamsters [25] and after 8 weeks in rats [36]; however, hypercholesterolemia needs a longer treatment time to show clearly significant increases—around 13 weeks of a cafeteria diet [16]. Changes in glucose tolerance start at week 7, according to Sampey and colleagues [12], with an increase in glycemia after 15 weeks. Plasma indicators of inflammation were not observed after 8 weeks of a cafeteria diet [36], but they were clear after 13 weeks [37]. Therefore, according to these references, the effects of GSPE on body weight could be clearly seen very early after the beginning of a cafeteria diet, as previously discussed. However, changes in plasma lipids, inflammation and glucose homeostasis need at least seven–eight weeks to clearly disrupt metabolic homeostasis. Therefore, we could not evaluate GSPE properly in our PRE-CAF2 group, because we only saw tendencies that were due to the cafeteria diet. In the case of the PRE-CAF1 group, there were also some protective tendencies associated with the effect of GSPE; however, 17 weeks of a cafeteria diet was too great of a challenge to fight against the negative effects of the diet. A similar situation was found in the CORR-CAF treatment, which also showed a limited effect similar to the pre-treatments. In this case, cafeteria-disrupting effects were clearly shown at the beginning of the treatment (15 weeks). Several GSPE corrective treatments applied to rats on cafeteria diets for similar periods of time but with lower doses have shown some level of effectiveness but they have been applied after shorter cafeteria diet challenges. Regarding inflammation, only a dose of 50 mg GSPE/kg b.w. reduced CRP plasma levels in the short treatment without changes in plasmatic TNF-α [26]. After 11 weeks of the cafeteria diet, 25 mg GSPE/kg b.w. improved triglyceridemia, cholesterolemina and glucose homeostasis [38], and a similar effect was found after 13 weeks of the cafeteria diet [14,16]. From our treatments, only the simultaneous intermittent treatment (SIT-CAF) managed to prevent the increase in the amount of triglycerides in plasma and liver, plasma cholesterol, and TNF-α. It also showed a strong tendency to improve glucose homeostasis, suggesting that it is necessary to administer the lipotoxic-inducing agent at early stages, to clearly avoid this lipotoxicity. Some preventive studies of GSPE have been assessed at lower doses. The dose of 25 mg/kg b.w. improved the HOMA index at week 9 [7]. During a 15-week treatment, LPS plasma levels showed a tendency to increase under the cafeteria treatment, and a dose of 25 mg GSPE/kg b.w. tended to keep them the same as the control group [39].

Summarizing all the considered aspects regarding the different administration time points of a GSPE dose of 500 mg/kg b.w., we can define the most optimal approach for using GPSE as an agent to avoid unhealthy states related to obesity. We have previously discarded the use of higher doses because they can produce negative effects [29], some of them related to desensitization to enterohomone signaling [40]. Dietary doses need to be used from the beginning of the challenge to produce the most effective results [7,39]. We found that the dose assessed in this work was very effective when taken as a simultaneous intermittent treatment. Because the PRE-CAF2 treatment remained effective for at least seven weeks, we suggest an intermittent dosage pattern to avoid possible desensitizing effects, but which could be more spaced in time than the SIT-CAF assessed here to try to obtain optimal effects with the lowest dose possible. This approach could be directly translated to the human population, because it is a product available for human consumption. However, the timing of the treatment and the optimal dose need to be adjusted too. 

## 5. Conclusions

In conclusion, a dose of 500 mg of GSPE/kg body weight administered following an intermittent dosage pattern, with an interval between doses longer than every other week, would be an optimal treatment against obesogenic metabolic challenges, such as a cafeteria diet.

## Figures and Tables

**Table 1:** Composition of meals offered.

	kJ/g	% Energy (CH)	% Energy (Protein)	% Energy (Lipid)
STD Chow	12.13	72.5	19.3	8.2
PRE-CAF1	20.73	52.0	14.1	33.9
PRE-CAF2-HFHS	17.66	77.3	16.2	6.5
PRE-CAF2-CAF	24.1	54.0	11.0	36.0

STD: standard chow provide to the control group; PRE-CAF1: cafeteria diet provide to the rats in the long-term study; PRE-CAF2-HFHS: High-Fat-High Sucrose diet provided during the 10 days with the GSPE (Grape-seed proanthocyanidins) treatment previous to the short-terms cafeteria intervention; PRE-CAF2-CAF: cafeteria diet provided to the rats in the short-term study .

**Table 2:** Morphometric and biochemical characteristics of the groups analyzed at 17 weeks.

Variable	STD	CAF	PRE-CAF1	SIT-CAF	CORR-CAF
Morphometric measurements					
*n*	9	10	10	7	9
Initial body weight(g)	220.7 ± 4.5	216.6 ± 3.5	214.2 ± 3.7	213.04 ± 5.2	219.6 ± 3.3
Final body weight (g)	273.7 ± 7.8	346.2 ± 12.0 *	316.8 ± 9.2	297.3 ± 9.8 ^#^	331.5 ± 12.6
mWAT (g)	4.2 ± 0.4	11.3 ± 1.3 *	9.5 ± 0.9	6.1 ± 0.7 ^#^	9.8 ± 1.0
oWAT (g)	7.5 ± 0.9	19.5 ± 2.0 *	15.9 ± 1.4	12.7 ± 1.4 ^#^	18.5 ± 2.1
rWAT (g)	3.8 ± 0.4	10.7 ± 1.2 *	9.8 ± 0.9	6.3 ± 0.6 ^#^	9.7 ± 1.1
Total visceral WAT (g)	15.4 ± 1.6	41.5 ± 3.8 *	35.1 ± 2.7	25.1 ± 2.6 ^#^	38.1 ± 3.9
BAT (g)	0.5 ± 0.1	1.0 ± 0.1 *	0.9 ± 0.1	0.8 ± 0.1 ^#^	1.0 ± 0.1
% visceral adiposity	5.6 ± 0.5	11.8 ± 0.8 *	11.0 ± 0.6	8.4 ± 0.6 ^#^	11.3 ± 0.8
% total adiposity (14 week)	9.0 ± 0.9	24.7 ± 1.6 *	21.1 ± 1.6	14.5 ± 1.6 ^#^	
Liver (g)	7.7 ± 0.3	8.9 ± 0.4 *	8.5 ± 0.3	8.5 ± 0.6	9.3 ± 0.3
Pancreas (g)	1.1 ± 0.1	1.0 ± 0.1	1.2 ± 0.1	1.0 ± 0.1	1.1 ± 0.2
Spleen (g)	0.5 ± 0.1	0.6 ± 0.0	0.6 ± 0.0	0.6 ± 0.1	0.7 ± 0.0
Thymus (g)	0.2 ± 0.0	0.3 ± 0.0 *	0.3 ± 0.0	0.3 ± 0.1	0.3 ± 0.0
Kidney (g)	1.5 ± 0.0	1.7 ± 0.1 *	1.6 ± 0.0	1.7 ± 0.1	1.8 ± 0.0
Plasma biochemical parameters					
Triglycerides (mM)	0.41 ± 0.07	0.56 ± 0.06	0.42 ± 0.05	0.35 ± 0.05 ^#^	0.45 ± 0.06
Cholesterol (mM)	1.6 ± 0.16	2.1 ± 0.17 *	1.9 ± 0.14	1.5 ± 0.13 ^#^	1.7 ± 0.13
Free Fatty Acids (mM)	0.21 ± 0.04	0.42 ± 0.09	0.33 ± 0.03	0.24 ± 0.09	0.44 ± 0.11
Glucose (mM)	8.9 ± 0.7	10.2 ± 4.0	10.0 ± 6.0	9.7 ± 6.0	10.4 ± 5.0
Insulin (µg/L)	5.6 ± 0.9	5.0 ± 0.8	6.4 ± 1.0	3.8 ± 0.8	9.7 ± 1.2

STD: lean rats fed a standard chow diet; CAF: rats fed a cafeteria diet; PRE-CAF1: rats receiving GSPE preventive treatment 10 days before the cafeteria intervention started; SIT-CAF: rats receiving GSPE treatment simultaneously and intermittently with the cafeteria diet every other week; CORR-CAF: rats receiving GSPE corrective treatment during the last 15 days of the cafeteria intervention; BAT: brown adipose tissue; GSPE: grape seed proanthocyanidin extract; mWAT: mesenteric white adipose tissue; oWAT: periovaric white adipose tissue; rWAT: retroperitoneal white adipose tissue; TAGs: triglycerides; % total adiposity was measured by NMR. Values are means ± SEM. * *p* < 0.05 compared to STD rats. ^#^ *p* < 0.05 compared to the CAF group.

**Table 3:** Morphometric and biochemical characteristics of the short treatment groups.

Variable	CAF	PRE-CAF2
Morphometric variables		
*n*	7	7
Initial body weight(g)	225.6 ± 2.2	223.4 ± 2.1
Final body weight (g)	307.7 ± 6.7	281.4 ± 4.0 ^#^
mWAT (g)	9.3 ± 1.0	6.0 ± 0.6 ^#^
oWAT (g)	17.3 ± 1.1	14.5 ± 1.0
rWAT (g)	12.7 ± 0.9	9.8 ± 0.6 ^#^
Total WAT (g)	40.2 ± 2.1	31.0 ± 1.7 ^#^
BAT (g)	0.9 ± 0.1	0.8 ± 0.0 ^#^
% visceral adiposity	13.1 ± 0.5	11.0 ± 0.8 ^#^
% total adiposity	24.7 ± 1.2	19.5 ± 1.4 ^#^
Plasma biochemical parameters		
Triglycerides (mM)	0.49 ± 0.04	0.40 ± 0.03
Cholesterol (mM)	3.09 ± 0.07	2.80 ± 0.12
Free Fatty Acids (mM)	0.28 ± 0.03	0.32 ± 0.05
Glucose (mM)	10.2 ± 0.3	10.4 ± 0.4
Insulin (µg/L)	5.1 ± 0.4	3.9 ± 0.7

CAF: rats fed a cafeteria diet; PRE-CAF2: rats receiving GSPE preventive treatment for 10 days together with a high fat/high sucrose diet followed by the cafeteria diet; BAT: brown adipose tissue; GSPE: grape seed proanthocyanidin extract; mWAT: mesenteric white adipose tissue; oWAT: periovaric white adipose tissue; rWAT: retroperitoneal white adipose tissue; TAGs: triglycerides; % total adiposity was measured by NMR. Values are means ± SEM. ^#^ *p* < 0.05 compared to the CAF group.
